# Large insertion in radish *GRS1* enhances glucoraphanin content in intergeneric hybrids, *Raphanobrassica* (*Raphanus sativus* L. x *Brassica oleracea* var. *acephala*)

**DOI:** 10.3389/fpls.2023.1132302

**Published:** 2023-06-06

**Authors:** Ryota Endo, Hiroshi Chikano, Etsuko Itabashi, Mitsuyo Kawasaki, Takayoshi Ohara, Tomohiro Kakizaki

**Affiliations:** ^1^ Agricultural and Bio Resource Development Department, Innovation Division, KAGOME CO., LTD., Nasushiobara, Japan; ^2^ Institute of Vegetable and Floriculture Science, National Agriculture and Food Research Organization, Tsu, Japan

**Keywords:** glucosinolate, radish, kale, intergeneric hybrid, *Raphanobrassica*, *GLUCORAPHASATIN SYNTHASE 1*, sulforaphane, glucoraphanin

## Abstract

Glucosinolates (GSLs), precursors of isothiocyanates (ITCs), are present in *Brassicaceae* plants have been found to have health benefits. Sulforaphane (4-(methylsulfinyl)butyl ITC) is an ITC stored in the form of 4-(methylsulfinyl)butyl GSL (glucoraphanin, 4MSOB) in *Brassica* vegetables, such as broccoli and kale. Sulforaphane activates Nrf2 expression, a transcription factor responsible for inducing physiological activities such as detoxification in the human body, and it represents a functional component unique to cruciferous vegetables. *Raphanobrassica* is an inter-generic hybrid between radish and kale, and it contains a high amount of 4MSOB. However, *Raphanobrassica* contains as much 4-methylsulfinyl-3-butenyl GSL (glucoraphenin, 4MSO3B) as it does 4MSOB. GLUCORAPHASATIN SYNTHASE 1 (GRS1) is an enzyme present in radish that synthesizes 4-methylthio-3-butenyl GSL (glucoraphasatin, 4MT3B), a precursor of 4MSO3B, using 4-(methylthio)butyl GSL (glucoerucin, 4MTB) as a substrate. Since the precursor of 4MSOB is also 4MTB, it was considered that both 4MSOB and 4MSO3B accumulate owing to competition in *Raphanobrassica*. We hypothesized that owing to the impaired function of GRS1 in *Raphanobrassica*, it may be possible to breed *Raphanobrassica* cultivars containing a high 4MSOB content. In this study, we generated *Raphanobrassica* populations with functional and defective *GRS1* and compared the GSL composition in the two populations using high-performance liquid chromatography. The mean 4MSOB content in leaves of the defective-type populations was higher than that in the functional-type population, and the defective/functional ratio ranged from 2.02 to 2.51-fold, supporting this hypothesis. Furthermore, leaves, flower buds, stems, and roots contained higher amounts of 4MSOB in the defective population than in the functional population. The leaf 4MSOB content of defective *Raphanobrassica* grown in this study was comparable to that of previously studied vegetables (such as broccoli sprouts) with high 4MSOB content. *Raphanobrassica* with defective *GRS1* represents a new leafy vegetable with high 4MSOB content which exhibits anti-cancerous and anti-inflammatory potentials.

## 1 Introduction

More than 500 plant species, primarily cruciferous plants, have been found to contain glucosinolates (GSLs). The common structure of GSLs consists of a β-D-glucopyranose residue linked by a sulfur atom to a (Z)-*N*-hydroximinosulfate ester, plus a variable side chain derived from precursor amino acids (Fahey et al., 2001; Halkier and Gershenzon, 2006). Based on the type of amino acid from which they are produced, GSLs can be categorized as aliphatic, benzenic, and indolic GSLs. Methionine (or alanine, isoleucine, leucine, and valine as alternatives), phenylalanine, and tryptophan are precursors of aliphatic, benzenic, and indolic GSLs, respectively. When plant tissues are damaged by herbivores and/or during infection, GSLs are hydrolyzed by myrosinases to isothiocyanates, thiocyanates, nitriles, or epithionitriles, depending on pH and the presence of epithiospecifier protein (Bones and Rossiter, 1996; Rask et al., 2000).

4-(Methylsulfinyl)butyl GSL (glucoraphanin, 4MSOB), which is a precursor of sulforaphane, is an aliphatic GSL. Sulforaphane has several physiological activities, one of which involves the activation of the transcription factor NF-E2–related factor 2 (Nrf2) (Kensler et al., 2013; Yuanfeng et al., 2021). Nrf2 is a master regulator of detoxification and antioxidants, and it controls the expression of downstream antioxidant genes and phase II detoxification enzyme genes by activating the oxidation response element (Chen and Maltagliati, 2018). The health benefits of sulforaphane have been widely studied in humans; sulforaphane intake reduces the level of urinary 8-hydroxyguanosine, an oxidative stress marker, and it decreases gamma-glutamyl transpeptidase and alanine transaminase levels, which are indicators of liver dysfunction (Kikuchi et al., 2015). Cognitive function is improved by the simultaneous intervention of sulforaphane intake and brain training (Nouchi et al., 2021). In addition, sulforaphane intake mediates the excretion of mycotoxins and air pollutants and improves mild asthma symptoms (Kensler et al., 2005; Riedl et al., 2009; Egner et al., 2014).

**Figure 1:**
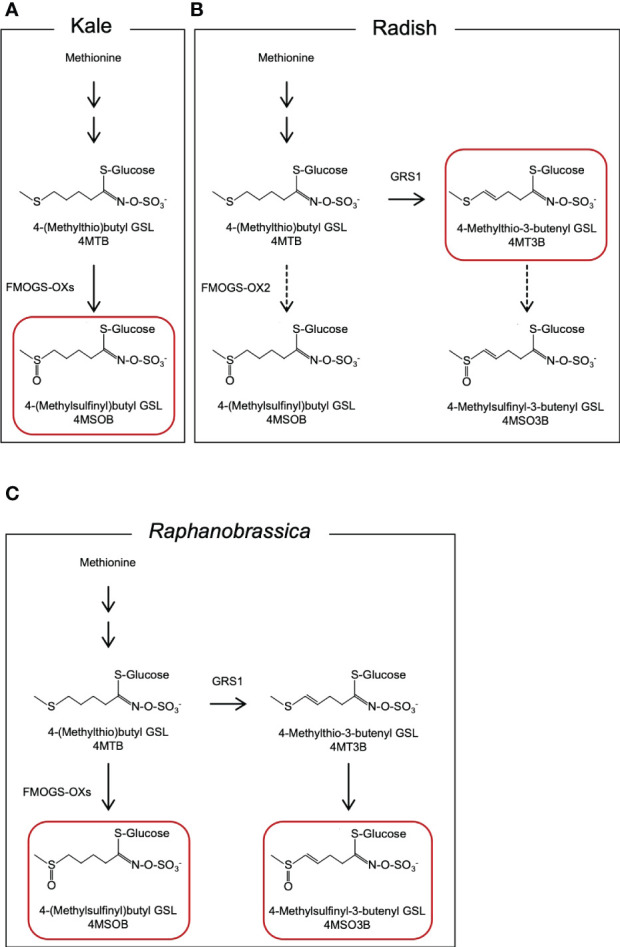
Presumed metabolic pathway of aliphatic GSLs in **(A)** kale, **(B)** radish, and **(C)** *Raphanobrassica* leaves. GSL, glucosinolate. The GSLs indicated by red boxes are the major GSLs in each species.

4MSOB accumulates in certain *Brassica oleracea* vegetables (2n = 2x = 18, CC genome; **Figure 1A**) (Cartea and Velasco, 2007). Broccoli (*B. oleracea* var. *italica*) contains the highest amount of 4MSOB among the currently evaluated *Brassica* vegetables. Farnham et al., (2004) reported that the average of 4MSOB concentration in 32 broccoli variety was 0.36 µmol·g^-1^ fresh weight and the range of 4MSOB was 0.24 to 1.85 µmol·g^-1^ fresh weight. It is known that 4MSOB accumulates in all tissues of broccoli and its content is particularly high in mature seeds and seedlings (Yagishita et al., 2019). 4MSOB is also known to be contained in red cabbage (Wermter et al., 2020). Several studies have attempted to increase the 4MSOB content in broccoli, and high 4MSOB-containing broccoli has been bred from crosses between a closely related wild species, *Brassica villosa*, and broccoli (Traka et al., 2013). Recently, it was suggested that MYB28 derived from *B. villosa* increases the transcription of genes that encode GSL biosynthesis enzymes in broccoli (Neequaye et al., 2022). Another 4MSOB-rich vegetable is allopolyploid *Raphanobrassica* (2n = 4x = 36, RRCC), an intergeneric hybrid of the genera *Raphanus* and *Brassica*. Initially, it was primarily used in cytogenetic studies to achieve the introduction of valuable traits (Karpechenko, 1924; McNaughton, 1973). *Raphanobrassica* contains 4MSOB, which is present in high concentrations in *B. oleracea*, and it contains 4-methylsulfinyl-3-butenyl GSL (glucoraphenin, 4MSO3B), which is in the leaves of *Raphanus* plants (Schutze et al., 1999; Niimi et al., 2015). The ratio of 4MSOB to 4MSO3B content in *Raphanobrassica* is lower; therefore, the development of a cultivar with a relatively higher 4MSOB content is desirable.

The radish *GLUCORAPHASATIN SYNTHASE 1* (*GRS1*) gene encodes a 2-oxoglutarate-dependent dioxygenase that presumably desaturates the 4-(methylthio)butyl GSL (glucoerucin, 4MTB) side chain (**Figure 1B**). A *GRS1* insertional mutants (*grs1*) are known for their high 4MTB accumulation (Kakizaki et al., 2017). Based on these facts, we hypothesized that metabolism would shift towards 4MSOB synthesis in radish lines containing the *grs1* allele as a parent of *Raphanobrassica* (**Figure 1C**). This would enable the breeding of *Raphanobrassica* cultivars with high 4MSOB content in the leaves. In this study, allodiploid *Raphanobrassica* (2n = 2x = 18, RC) plants were produced from a cross between kale and radish with heterozygous *GRS1*, and the correlation between *GRS1* genotype and 4MSOB content was analyzed.

## 2 Methods

### 2.1 Plant material and growth conditions

A previous study reported a Japanese radish (*Raphanus sativus* L.) landrace, ‘cv. Nishimachi-Riso’, which contains a *GRS1* mutation (Ishida et al., 2015). As the genotype of *GRS1* was not fixed within the ‘cv. Nishimachi-Riso’ population, the genotype of *GRS1* was determined using DNA markers (see 2.2 Genotyping) and plants carrying heterozygouse *GRS1* (*GRS1/grs1*) were designated as AKO lines. Kale (*Brassica oleracea* var. *acephala*) inbred line ‘KK45-2’ is a collard-type kale whose leaves contain 4MSOB used as the paternal parent. All the plants were grown in plastic pots (diameter of 210 mm) in a greenhouse. A Nippi-engei-baido (Nihon Hiryo Co.,Ltd, Gunma, Japan) based soil was used and 1/1000 HYPONeX (HYPONeX JAPAN CORP., LTD., Osaka, Japan) solution was applied once a week. To obtain hybrids, the anthers were removed from radish buds 1–3 d before flowering and were pollinated with kale pollen on the day of flowering. *Raphanobrassica* seeds were sown on September 5, 2020, and genomic DNA was purified from the true leaves two weeks later and used for genotyping. For genomic DNA purification, DNeasy 96 Plant Kit (QIAGEN, Venlo, Netherlands) was used. The plants were then planted in a field on August 31, 2020, with a gap of 35 cm between plants and 60 cm between rows, at the Institute of Vegetable and Floriculture Science (34°46′N, 136°25′E; Tsu, Mie, Japan).

### 2.2 Genotyping

**Figure 2:**
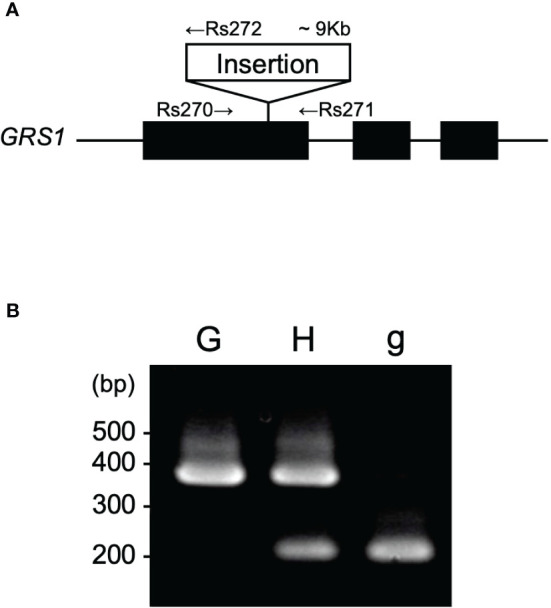
**(A)** Gene structure of *GRS1*. Black boxes represent exons. Arrows represent the position of primers for PCR. **(B)** Representative agarose gel pattern of amplified PCR product. “G”, “H”, and “g” indicate homozygote for *GRS1*, heterozygote, and homozygote for *grs1*, respectively. *GRS1, GLUCORAPHASATIN SYNTHASE 1*; PCR, polymerase chain reaction.

Previous studies have shown that insertion into the first exon of *GRS1* causes complete functional defects (Kakizaki et al., 2017). To detect the insertion into *GRS1*, primers were designed at the genomic positions shown in **Figure 2A**, and polymerase chain reaction (PCR) was used to amplify the region between Rs270 (5′-GCAGGAGAGGATGCTTGAAGG-3′) and Rs271 (5′-TGAAACCTTACCCCAAAACG-3′) for the functional type, Rs270 and Rs272 (5′-TCCAGGTTGGGATAGCTTGT-3′) for the defective type. PCR was performed under the following conditions: initial denaturation at 94°C for 1 min, cycling at 94°C for 15 s for heat denaturation, annealing at 60°C for 15 s, and extension at 72°C for 50 s for 32 cycles. The amplified PCR products were separated on a 2% agarose gel, and the functional and defective types were distinguished based on differences in fragment length (**Figure 2B**). To confirm that the kale genome was inherited by the hybrid, PCR was performed using primers Bo-Fw (5′-CTAGTATGAGGACTCGTTCAGTTACCTCCCTTAGCAGC-3′) and Bo-Rv (5′-GTTTCTTAGAATATGGTGATTGCTGGCTT-3′) to amplify *UDP-sulfoquinovose synthase* (LOC106306866), which is located on chromosome C1.

### 2.3 GSL analysis

**Figure 3:**
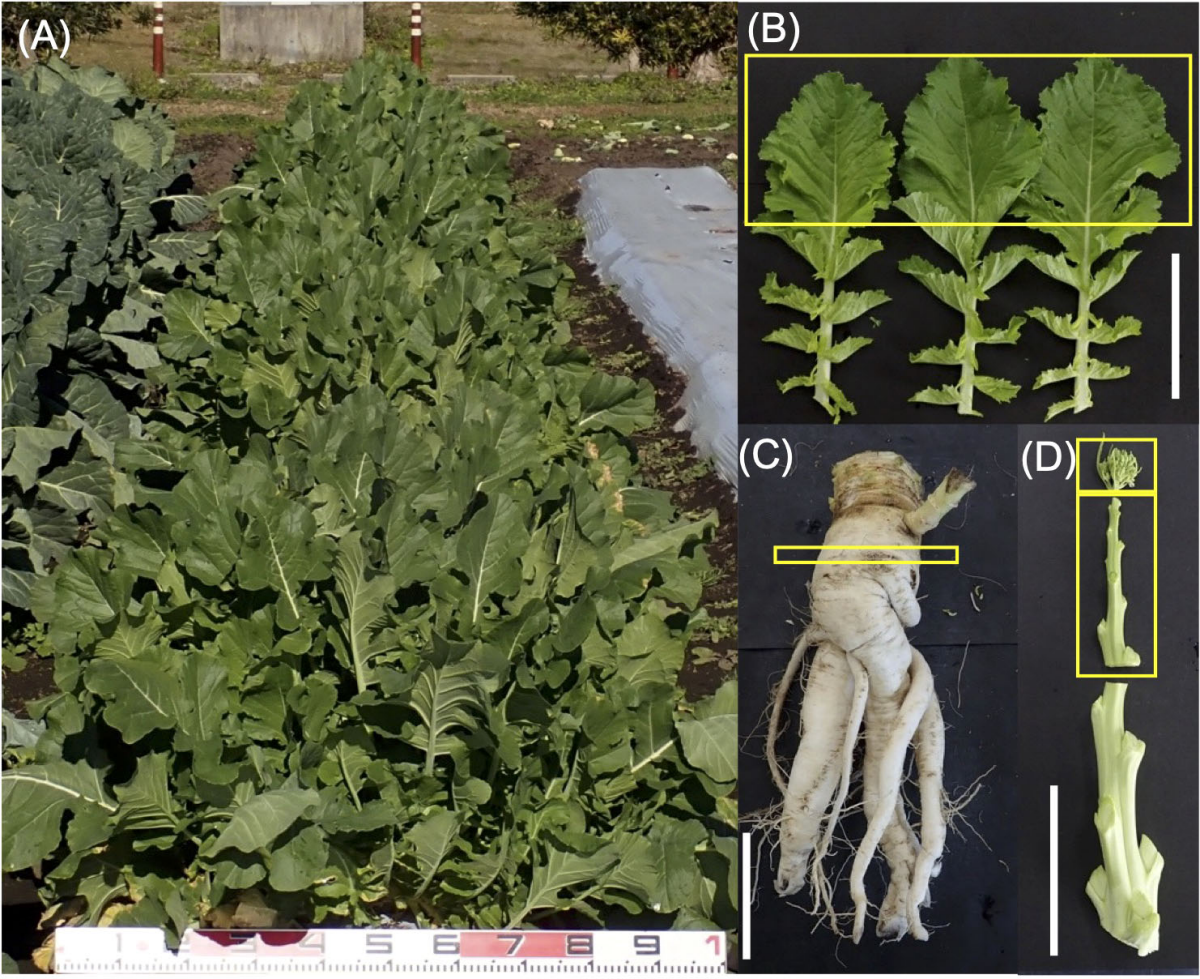
**(A)** Typical phenotypic characteristics of *Raphanobrassica* **(A)**. Plant parts used for glucosinolate analysis: **(B)** leaf, **(C)** root, **(D)** flower bud, and stem. Yellow boxes show a part used for high-performance liquid chromatography analysis. White bars indicate 10 cm.

Leaf, root, stem, and flower bud are used for GSL analysis (**Figure 3**). For leaf analysis, 10 cm from the tip of the 20 cm long leaf was collected, and the central vein was removed. Three leaves per plant were collected and analyzed as one bulk sample. For root analysis, a section was cut 5 cm below the stem/hypocotyl border in the form of a disk (thickness, 0.5–1.0 cm) and collected. For bud analysis, apical flower buds were collected. For stem analysis, a 10 cm section below the apical flower bud of the main stem, which was elongated after bolting, was collected.

**Figure 4:**
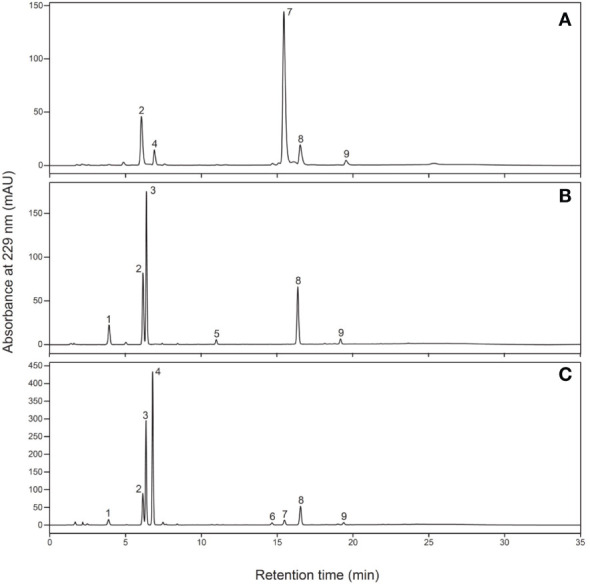
Typical chromatograms of desulfo-glucosinolates in **(A)** radish, **(B)** kale, and **(C)** *Raphanobrassica*. Peak 1, 3-(methylthio)propyl; peak 2, 2-propenyl (internal standard); peak 3, 4-(methylsulfinyl)butyl; peak 4, 4-methylsulfinyl-3-butenyl; peak 5, 4-methoxyindol-3-ylmethyl; peak 6, 4-(methylthio)butyl; peak 7, 4-methylthio-3-butenyl; peak 8, indol-3-ylmethyl; peak 9, 4-methoxyindol-3-ylmethyl.

After sampling, each part was frozen in liquid nitrogen and dried using a lyophilizer (Labconco, Kansas City, MO, USA). The dried samples were crushed using a multi-bead shocker (Yasui Machinery, Miyazaki, Japan); 0.1 g of ground samples were weighed, mixed with 4.8 mL of 80% methanol and 0.2 mL of 5 mM 2-propenyl GSL (Sigma-Aldrich, St. Louis, MO, USA) as an internal standard and shaken for 30 min at 25°C. After centrifugation at 3,000 rpm for 10 min, the supernatant was collected, GSLs were adsorbed onto DEAE-Sephadex A-25 (Sigma-Aldrich, St. Louis, MO, USA) and desulfonated using arylsulfatase (Type H-1, EC 3.1.6.1, Sigma-Aldrich, St. Louis, MO, USA) at 25°C for 18 h. Desulfo-GSL solutions eluted ion-exchange water were used as samples for high-performance liquid chromatography (HPLC) analysis (LC-20A; Shimadzu Corp., Kyoto, Japan). A reverse-phase column (COSMOSIL 5C18-II, 150 × 4.6 mm; Nacalai Tesque Inc., Kyoto, Japan) was used at 30°C and a flow rate of 1.5 mL/min. The mobile phase comprised 20% acetonitrile, and detection was performed using UV light with a wavelength of 229 nm. The GSL molecular species were estimated based on the retention time of the peaks according to our previous report (Ishida et al., 2015). The individual GSL contents were calculated by the ratios of the individual desulfo-GSL peak areas to the peak areas of an internal standard, 2-propenyl GSL (Sigma-Aldrich, St. Louis, MO, USA), and a response factor (The International Organization for Standardization, 1992). **Table 1** shows the list of GSLs analyzed, and **Figure 4** shows representative chromatograms of each desulfo-GSL molecular species detected using HPLC.

**Table 1:** Information of glucosinolates in the present study.

Peak number^a^	Retention time (min)	Chemical name	Trivial name	Abbreviation	Compound groups	Radish RR genome	Kale CC genome	Raphanobrassica RC genome
1	3.7	3-(Methylthio)propyl	Glucoiberin	3MSOP	Aliphatic		✓^c^	✓
2^b^	5.9	2-Propenyl	Sinigrin	2-Propenyl	Aliphatic	I.C.	I.C.	I.C.
3	6.2	4-(Methylsulfinyl)butyl	Glucoraphanin	4MSOB	Aliphatic		✓	✓
4	6.6	4-Methylsulfinyl-3-butenyl	Glucoraphenin	4MSO3B	Aliphatic	✓		✓
5	10.8	4-Methoxyindol-3-ylmethyl	4-Hydroxyglucobrassicin	4OH-I3M	Indolic		✓	
6	14.4	4-(Methylthio)butyl	Glucoerucin	4MTB	Aliphatic			✓
7	15.2	4-Methylthio-3-butenyl	Glucoraphasatin	4MT3B	Aliphatic	✓		✓
8	16.3	Indol-3-ylmethyl	Glucobrassicin	I3M	Indolic	✓	✓	✓
9	19.1	4-Methoxyindol-3-ylmethyl	4-Methoxyglucobrassicin	4MO-I3M	Indolic	✓	✓	✓

^a^Peak number is same as in **Figure 1**. ^b^2-Propenyl GSL is used for internal control (I.C.). ^c^✓ indicates GSL detected.

## 3 Results

### 3.1 Intergeneric cross between radish and kale

To generate intergeneric hybrids that harbored the *grs1* allele, we used a radish AKO line that was heterozygous for the *GRS1* gene (*GRS1*/*grs1*) as a seed parent. The kale KK45-2 line, containing 4MSOB in mature leaves, was used as the pollen parent. At the time of crossing, all stamens were removed from radish buds 1–2 d before flowering to avoid self-fertilization, and pollination was performed using kale pollen on the day of flowering. Twenty-one AKO plants were pollinated using KK45-2 pollen, and the pod formation rate varied from 0 to 0.24 among radish plants (**Table 2**). Similarly, there was a difference in the number of seeds per pod among the AKO plants. Among the 21 combinations, the seeds of four radish plants (AKO103, AKO108, AKO110, and AKO118) that yielded a large number of seeds were sown in petri dishes, and their germination rates and genotypes were investigated (**Table 3**). The germination percentage ranged from 71.4–97.1%, and 47–87 plants were obtained from each combination. To confirm that the obtained plants were hybrids, PCR was performed using primers specific for kale *UDP-sulfoquinovose synthase* (LOC106306866). This confirmed the inheritance of the kale genome. No amplification of the kale genome was detected in only four plants obtained from AKO110, and these plants were inbred radish plants. Genotyping using the *GRS1* marker (**Figure 2**) resulted in a 1:1 match for *GRS1* segregation in all combinations. No plants heterozygous for the *GRS1* marker were identified. Based on these results, we obtained hybrids of radish and kale, and *GRS1* segregation followed theoretical values. These data allowed us to evaluate the relationship between the *GRS1* genotype and 4MSOB quantity in the *Raphanobrassica* population.

**Table 2:** Comparative results of intergeneric cross between radish x kale.

♀:Radish line	♂:Kale line	Number of pollinations	Number of sillique developed	Number of seeds obtained	pod/pollinated flower	Seed/pollinated flower
AKO101	KK45-2	422	1	1	0.00	0.00
AKO102	KK45-2	1142	9	11	0.01	0.01
AKO103	KK45-2	595	143	114	0.24	0.19
AKO104	KK45-2	304	2	3	0.01	0.01
AKO105	KK45-2	525	5	5	0.01	0.01
AKO106	KK45-2	517	0	0	0.00	0.00
AKO107	KK45-2	331	0	0	0.00	0.00
AKO108	KK45-2	503	72	105	0.14	0.21
AKO109	KK45-2	694	10	15	0.01	0.02
AKO110	KK45-2	845	57	129	0.07	0.15
AKO111	KK45-2	520	1	1	0.00	0.00
AKO112	KK45-2	898	9	8	0.01	0.01
AKO113	KK45-2	352	0	0	0.00	0.00
AKO114	KK45-2	685	13	22	0.02	0.03
AKO115	KK45-2	311	0	0	0.00	0.00
AKO116	KK45-2	212	0	0	0.00	0.00
AKO117	KK45-2	594	1	1	0.00	0.00
AKO118	KK45-2	515	109	175	0.21	0.34
AKO119	KK45-2	310	0	0	0.00	0.00
AKO120	KK45-2	409	4	4	0.01	0.01
AKO121	KK45-2	19	0	0	0.00	0.00

**Table 3:** Germination rate and segregation ratio of *GRS1* in allodiploid *Raphanobrassica*.

Cross combination	Number of seeds	Germination percentage (%)	Plants obtained	Absence of kale marker^a^	Number of hybrid	Genotype with *GRS1* marker			
						*GRS1*	*grs1*	*GRS1/grs1*	χ^2^ (1:1)^b^
AKO103 x KK45-2	88	86.4	47	0	47	18	29	0	2.574
AKO108 x KK45-2	70	97.1	55	0	55	29	26	0	0.164
AKO110 x KK45-2	112	71.4	72	4	68	31	37	0	0.529
AKO118 x KK45-2	113	85.0	87	0	87	46	41	0	0.287

a) UDP-sulfoquinovose synthase (LOC106306866), C01.

b) chi-squared = 3.841, *df* = 1, p = 0.05.

### 3.2 Suppression of *GRS1* function increased 4MSOB content in the intergeneric hybrid

**Figure 5:**
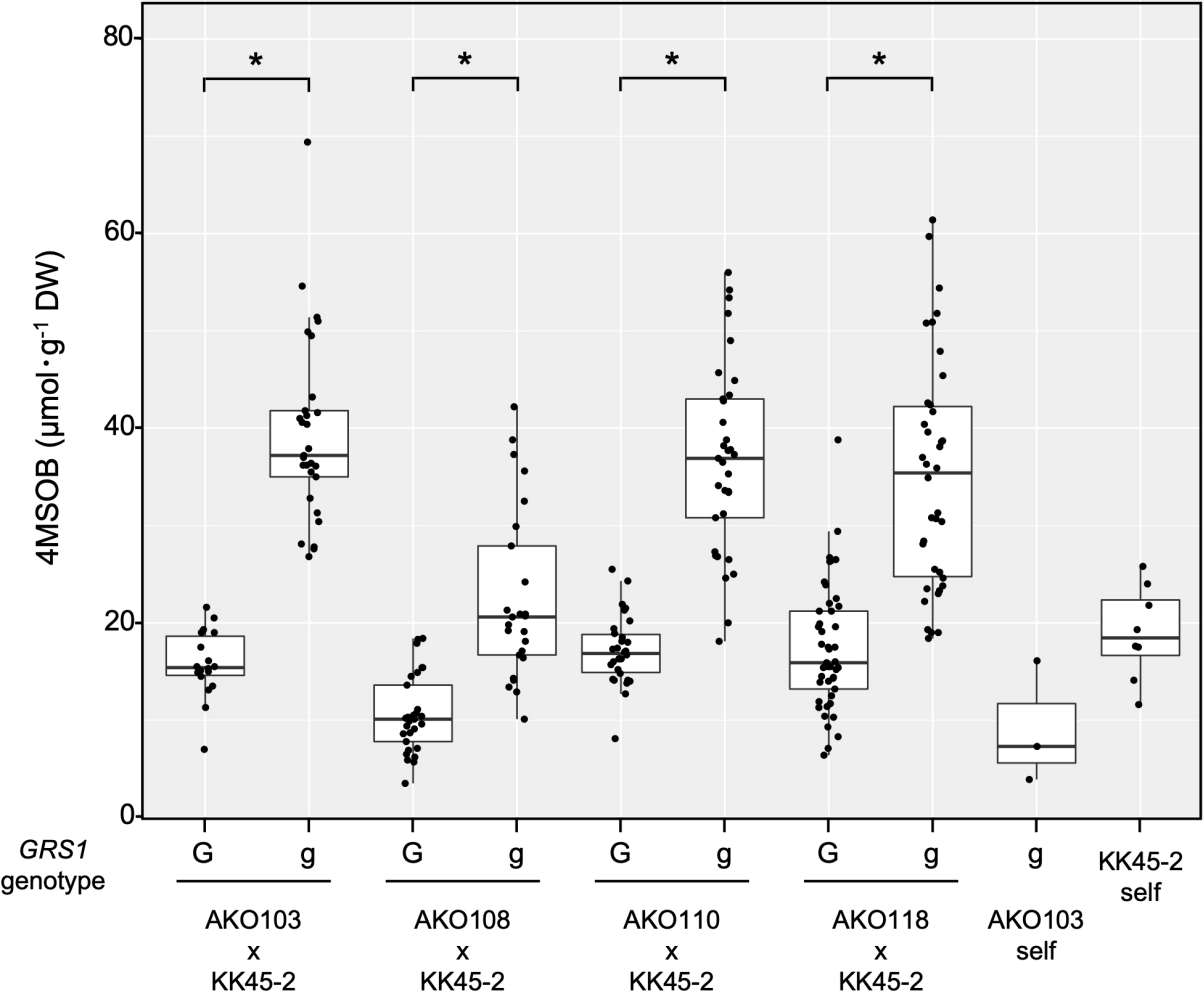
Distribution of leaf 4SMOB content in intergeneric hybrids and parent lines. The genotype of *GRS1* is indicated by “G” for functional and “g” for null allele. Asterisks above the bars indicate significant differences (p < 0.01) between the genotypes of *GRS1* as determined *via* the Welch’s t-test. *GRS1, GLUCORAPHASATIN SYNTHASE 1*.

**Figure 6:**
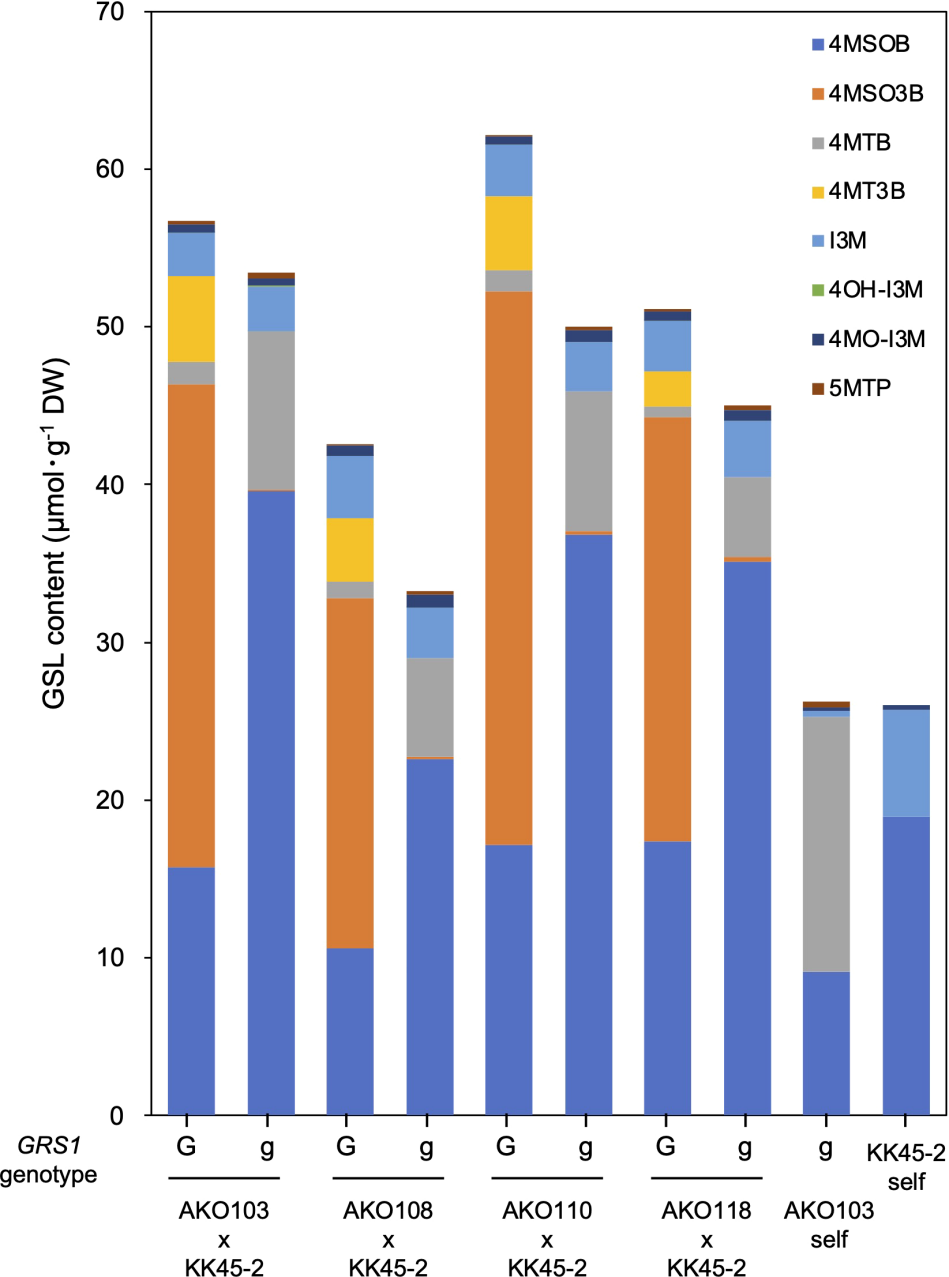
GSL composition and content in *Raphanobrassica* leaf. The genotype of *GRS1* is indicated by “G” for functional and “g” for null allele. *GRS1, GLUCORAPHASATIN SYNTHASE 1*; GSL, glucosinolate. 4MSOB, 4-(methylsulfinyl)butyl GSL; 4MSO3B, 4-methylsulfinyl-3-butenyl GSL; 4MTB, 4-(methylthio)butyl GSL; 4MT3B, 4-methylthio-3-butenyl GSL; I3M, indol-3-ylmethyl GSL; 4OH-I3M, 4-methoxyindol-3-ylmethyl GSL, 4MO-I3M, 4-methoxyindol-3-ylmethyl GSL; 5MTP, 5-(methylthio)pentyl GSL.

The obtained hybrids were planted in a field on September 5, 2020, and the GSL composition in the true leaves (20 cm length) was analyzed 72 d after planting. Growth was vigorous, and leaf shape was similar to that of radish (**Figures 3A, B**). No plants with pollen fertility were observed for any of the cross combinations. The root shape was snarled and the main root branched into several branches (**Figure 3C**). The timing of bolting was earlier than that of the parents, and the shape of the flower buds was similar to that of kale (**Figure 3D**). The 4MSOB content in the true leaves of intergeneric hybrids and parent plants is shown in **Figure 5**. Notably, in all cross combinations, the *grs1*-type had a 4MSOB content approximately twice as high as that of functional *GRS1* hybrids (**Table 4**). Furthermore, these *grs1*-type plants had higher 4MSOB concentrations than those in kale KK45-2 and radish AKO103 (*grs1*/*grs1*) plants. There were significant differences in the concentration of 4MSOB between *GRS1* genotypes, but even within the same *GRS1* genotype the concentration of 4MSOB varied widely (**Figure 5**). The average of 4MSOB in four lines in *GRS1*-type and *grs1*-type were 15.5 ± 0.5 and 34.1 ± 1.0 µmol·g^-1^ dry weight in leaves, respectively. The highest 4MSOB concentration was 69.4 µmol·g^-1^ dry weight. In *grs1*-type *Raphanobrassica*, 4MSO3B was almost undetectable and the accumulation of 4MTB was detected (**Figure 6**). The contents of indolic GSLs, such as Indol-3-ylmethyl GSL (glucobrassicin, I3M), were not affected by the *GRS1* mutation.

**Table 4:** Relationship between *GRS1* genotype and glucosinolate content.

Cross combination	*GRS1* genotype	Number of plants	µmol·g^-1^ dry weight		4MSOB ratio^a^
			4MSOB	4MSO3B	
AKO103 x KK45-2	*GRS1*	18	15.8 ± 0.8^bc^	30.6 ± 1.1^b^	2.51
	*grs1*	29	39.6 ± 1.7^a^	0.04 ± 0.3^d^	
AKO108 x KK45-2	*GRS1*	29	10.6 ± 0.7^c^	22.2 ± 1.0^c^	2.13
	*grs1*	25	22.6 ± 1.7^b^	0.2 ± 0.1^d^	
AKO110 x KK45-2	*GRS1*	30	17.2 ± 0.6^b^	35.1 ± 1.1^a^	2.14
	*grs1*	33	36.8 ± 1.7^a^	0.3 ± 0.1^d^	
AKO118 x KK45-2	*GRS1*	45	17.4 ± 1.0^b^	26.9 ± 1.2^b^	2.02
	*grs1*	38	35.1 ± 1.9^a^	0.2 ± 0.05^d^	
AKO103 S1	*grs1*	3	9.1 ± 3.0	not detected	
KK45-2 S1	–	8	19.0 ± 1.6	not detected	

Glucosinolate content is indicated as averages ± SE. Values given are μmol g^-1^ dry weight.

Same letters indicate no significant difference (Tukey-Kramer HSD test, P < 0.05).

^a^ 4MSOB ratio of *GRS1* type to *grs1* type as 1 for hybrids resulting from the same cross combination.

### 3.3 GSL content in roots, flower buds, and stems was affected by GRS1 function

**Figure 7:**
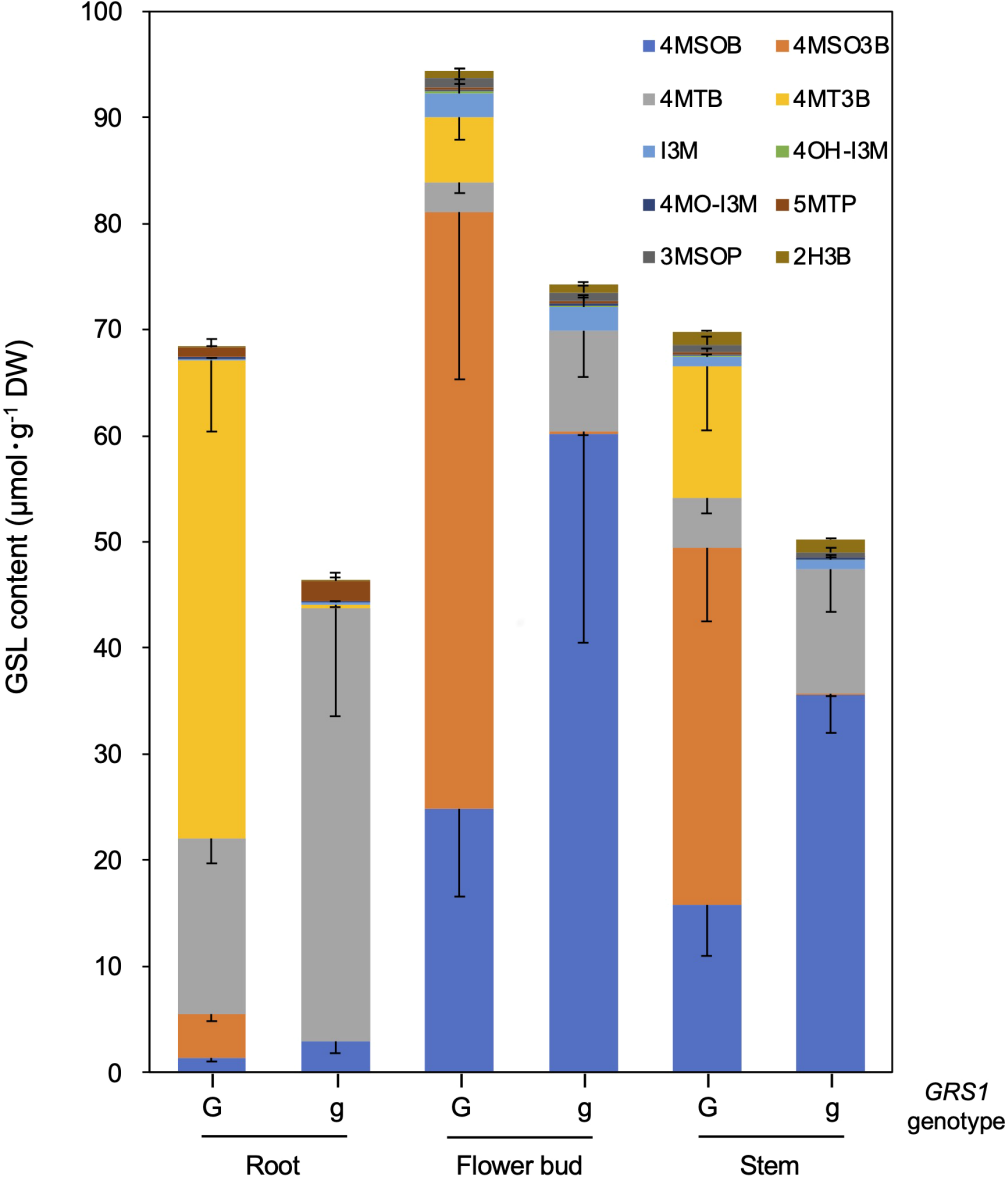
GSL composition and content in root, flower bud, and stem in *Raphanobrassica*. The genotype of *GRS1* is indicated by “G” for functional and “g” for null allele. Error bars represent standard deviation. *GRS1, GLUCORAPHASATIN SYNTHASE 1*; GSL, glucosinolate. 4MSOB, 4-(methylsulfinyl)butyl GSL; 4MSO3B, 4-methylsulfinyl-3-butenyl GSL; 4MTB, 4-(methylthio)butyl GSL; 4MT3B, 4-methylthio-3-butenyl GSL; I3M, indol-3-ylmethyl GSL; 4OH-I3M, 4-methoxyindol-3-ylmethyl GSL, 4MO-I3M, 4-methoxyindol-3-ylmethyl GSL; 5MTP, 5-(methylthio)pentyl GSL; 3MSOP, 3-(methythio)propyl GSL; 2H3B, 2-hydroxy-3-butenyl GSL.

The *grs1* mutation increased 4MSOB concentration by approximately 2-fold in the leaves of the hybrids. Next, we analyzed the GSL profiles in various edible plant parts such as roots, flower buds, and stems (**Table 5**). The 4MSOB content was highest in the flower buds, followed by stems and roots. The *grs1* mutation increased the concentration of 4MSOB by more than 2-fold in flower buds and stems, similar to that in leaves. The major GSLs in the roots were 4MT3B and 4MTB, whereas those in the flower buds and stems were 4MSO3B and 4MSOB (**Figure 7**). 2-Hydroxy-3-butenyl GSL (progoitrin, 2H3B) and I3M, which are rarely detected in the roots, were detected in flower buds and stems. Regarding the total GSL, the *GRS1-*type plants had a higher content than that in the *grs1*-type, similar to that in the leaves. The total GSL content was highest in flower buds for both *GRS1* and *grs1*-types, followed by roots and stems.

**Table 5:** Glucosinolate content in different organ of *Raphanobrassica*.

Cross combination	Organ	*GRS1* genotype	Number of plants	µmol·g^-1^ dry weight		4MSOB ratio^a^
				4MSOB	4MSO3B	
AKO103 x KK45-2	Root	*GRS1*	10	1.4 ± 0.1^d^	4.1 ± 0.2^c^	2.07
		*grs1*	10	2.9 ± 0.4^d^	not detected	
AKO110 x KK45-2	Flower bud	*GRS1*	10	24.8 ± 2.6^bc^	56.2 ± 5.0^a^	2.42
		*grs1*	10	60.1 ± 6.2^a^	0.2 ± 0.1^c^	
AKO110 x KK45-2	Stem	*GRS1*	10	15.8 ± 1.5^c^	33.6 ± 2.2^b^	2.25
		*grs1*	10	35.6 ± 1.1^b^	0.1 ± 0.1^c^	

Glucosinolate content is indicated as averages ± SE. Values given are μmol g^-1^ dry weight.

Same letters indicate no significant difference (Tukey-Kramer HSD test, P < 0.05).

^a^ 4MSOB ratio of *GRS1* type to *grs1* type as 1 for hybrids resulting from the same cross combination.

## 4 Discussion

### 4.1 An intergeneric hybrid between radish and kale

*Raphanobrassica*, an intergeneric hybrid between the genera *Raphanus* and *Brassica*, contains large amounts of 4MSOB and 4MSO3B (Schutze et al., 1999). Lyophilized powder derived from *Raphanobrassica* inhibits *Helicobacter pylori*-induced gastritis in Mongolian gerbils (Yamada et al., 2014). Although *Raphanobrassica* has useful properties, the hybridization rate between *Raphanus* and *Brassica* is considerably low. In 1973, McNaughton reported that the number of seeds per pollinated flower between *R. sativus* and *B. oleracea* is between 0.38 and 0.4 (McNaughton, 1973). This value is comparable to that of the most efficient combination in the crossing experiment of the present study (AKO118 × KK45-2, 0.34). Further, the formation rates of hybrids differ between varieties (Kakizaki, 1925). This phenomenon is known as the “hybridization barrier” and is divided into two types: pre-zygotic and post-zygotic barriers. The pre-zygotic barrier is caused mainly by defects in fertilization, such as interspecific incompatibility and defects in pollen tube guidance (Dresselhaus and Marton, 2009). The post-zygotic barrier includes hybrid embryo breakdown and hybrid sterility. Candidate genes or quantitative trait loci responsible for these barriers have been cloned (Udagawa et al., 2010; Tonosaki et al., 2013). In the present study, the hybridization rates were markedly different between AKO plants originating from the same radish ‘cv. Nishimachi-Riso’ (**Table 2**). It is not known whether the barriers observed in the present study are pre- or post-zygotic, but differences in their degree within the same species may provide good material for genetic analysis.

### 4.2 GSLs in *Raphanobrassica*

The hybridization between a radish and Chinese cabbage produces a cultivar called ‘Baemoochae’ (Lee et al., 2011). Baemoochae contains GSL molecular species present in both radish and Chinese cabbage. However, the total GSL content is similar to that of both parents (Nugroho et al., 2020). In contrast, the total GSL content of *Raphanobrassica* produced in this study was higher than that of either parent, suggesting that the entire GSL synthesis pathway was activated. It is reported that the GSL synthesis gene expression is elevated in resynthesized *Brassica* allotetraploids compared with that in their diploids (Zhang et al., 2015). Further studies are needed to determine whether the increased 4MSOB in *Raphanobrassica* developed in this study is solely due to mutations in *GRS1* or is also influenced by the increased expression of other biosynthetic enzyme genes. GSLs are hydrolyzed by myrosinases to not only ITCs but other products (thiocyanates, nitriles, or epithionitriles) depending on pH and the presence of epithiospecifier protein (Bones and Rossiter, 1996). Distribution of degradated products vary widely between plant species. *B. oleracea* has a higher proportion of nitriles and epithionitriles than ITCs, whereas *R. sativus* has a higher proportion of ITCs (Cole, 1976). Therefore, analysis of the abundance ratios of degradation products in *Raphanobrassica*, which has both genomes, is of great importance for its use as a functional vegetable.

### 4.3 Effects of *grs1* mutation on the GSL synthesis pathway

Hybrids containing *grs1* showed almost no 4MT3B or 4MSO3B content in their leaves (**Table 4** and **Figure 6**). This result supports the hypothesis that the introduction of *grs1* into *Raphanobrassica* enhances 4MTB utilization in 4MSOB synthesis and increases the 4MSOB content. However, in all cross combinations, the total content of each GSL in the population possessing *grs1*-type tended to be lower than that in *GRS1-*type plants (**Figure 6**). Therefore, increased 4MSOB content in plants lacking GRS1 function may cause feedback inhibition of FMO. For example, YUCCA, an enzyme in the auxin synthesis pathway, also belongs to the monooxygenase family, similar to FMO. *YUCCA* transcription levels are negatively regulated by the synthetic product auxin (Suzuki et al., 2015). If FMO activity and transcription levels are negatively regulated by 4MSOB, understanding the underlying mechanisms and applying them in breeding could lead to higher 4MSOB content.

### 4.4 Potential and challenges of using *Raphanobrassica* as a high-4MSOB containing vegetable

4MSOB is present in the genera *Brassica*, *Eruca, and Raphanus* (Ciska et al., 2000; Ishida et al., 2014). A study in 1992 reported that sulforaphane (ITC derived from 4MSOB) in broccoli functions as a major inducer of anti-carcinogenic defense enzymes. Consequently, the recognition of broccoli as a representative vegetable containing 4MSOB has increased, which has prompted research in various fields. To develop broccoli varieties with high 4MSOB content, *MYB28* of the wild species *B. villosa* (*BvMYB28*) has been introduced into cultivated species, and the F_1_ hybrid Beneforté^®^ has been cultivated (Traka et al., 2013). In *Arabidopsis*, *AtMYB28* positively regulates aliphatic GSL biosynthesis (Sonderby et al., 2007). The expression of several aliphatic GSL genes is elevated in broccoli, which is homozygous for *BvMYB28*. The 4MSOB concentration in the homozygous *BvMYB28* inbred broccoli line is 20 µmol·g^-1^ dry weight in floret (Neequaye et al., 2022). In contrast, the average value of 4MSOB in the leaves of *Raphanobrassica* with *grs1* grown in this study was 34.1 µmol·g^-1^ dry weight (**Table 4**). Although simple comparisons cannot be made because the analyzed sites contained the highest contents in adult plants and the content varies with the growing region and cultivation method, the variety with defective GRS1 in *Raphanobrassica* may be used in various cuisines as a new leafy vegetable that contains as much 4MSOB in broccoli Beneforté^®^. Compared with kale, the taste of *Raphanobrassica* leaves is softer and less gruel-like, making it suitable for a variety of dishes such as stir-fry and salads. The low hybridization affinity between radish and kale and the inability to ensure a commercial level of seed production represent issues that need to be addressed to popularize the variety in the future. However, chromosome doubling may be used to breed seed-fertile allotetraploid *Raphanobrassica* (Niimi et al., 2015).

## Data availability statement

The original contributions presented in the study are included in the article/**Supplementary Material**, further inquiries can be directed to the corresponding author/s.

## Author contributions

TK and HC supervised and conceived the project. RE and TK wrote the manuscript. EI, MK, and TO supported the experiments and revised the manuscript. TK and RE cultivated the plants and evaluated the GSLs. All authors contributed to the article and approved the submitted version.
